# Whole-exome sequencing of selected bread wheat recombinant inbred lines as a useful resource for allele mining and bulked segregant analysis

**DOI:** 10.3389/fgene.2022.1058471

**Published:** 2022-11-22

**Authors:** Salvatore Esposito, Nunzio D’Agostino, Francesca Taranto, Gabriella Sonnante, Francesco Sestili, Domenico Lafiandra, Pasquale De Vita

**Affiliations:** ^1^ Research Centre for Cereal and Industrial Crops (CREA-CI), CREA—Council for Agricultural Research and Economics, Foggia, Italy; ^2^ Department of Agricultural Sciences, University of Naples Federico II, Portici, Italy; ^3^ Institute of Biosciences and Bioresources (CNR-IBBR), Bari, Italy; ^4^ Department of Agriculture and Forest Sciences (DAFNE), University of Tuscia, Viterbo, Italy

**Keywords:** wheat, exome capture, target-enrichment, recombinant inbred lines, bulked segregant analysis (BSA), single nucleotide polymorphisms (SNPs)

## Abstract

Although wheat (*Triticum aestivum* L.) is the main staple crop in the world and a major source of carbohydrates and proteins, functional genomics and allele mining are still big challenges. Given the advances in next-generation sequencing (NGS) technologies, the identification of causal variants associated with a target phenotype has become feasible. For these reasons, here, by combining sequence capture and target-enrichment methods with high-throughput NGS re-sequencing, we were able to scan at exome-wide level 46 randomly selected bread wheat individuals from a recombinant inbred line population and to identify and classify a large number of single nucleotide polymorphisms (SNPs). For technical validation of results, eight randomly selected SNPs were converted into Kompetitive Allele-Specific PCR (KASP) markers. This resource was established as an accessible and reusable molecular toolkit for allele data mining. The dataset we are making available could be exploited for novel studies on bread wheat genetics and as a foundation for starting breeding programs aimed at improving different key agronomic traits.

## Introduction

Bread wheat (*Triticum aestivum* L. 2n = 6x = 42, AABBDD) is a major staple crop that provides about 20% of daily calories and 21% of protein needs consumed by the world population (FAO 2017, http://www.fao.org/3/a-i6583e.pdf). However, to meet global food demand without expanding acreage, wheat grain production is projected to increase by at least 50% within the next few decades (Tshikunde et al., 2019). This means that the average annual genetic gain of wheat is expected to increase 1.0%–1.7% *per* year, reaching global production of 1 billion tons in 2050 (Le Mouël et al., 2017). Considering also the need to adopt environmentally sustainable agronomic practices capable of mitigating the effect of climate change on soil degradation and water scarcity (https://www.ipcc.ch/report/srccl/), the next challenges appear to be very demanding for wheat breeders and scientists (De Vita and Taranto, 2019; Senapati et al., 2019; Xiong et al., 2021; The grand challenge of breeding by design, 2022). In addition, the genomic resources of wheat are scarce compared with those of other cereals such as rice and maize, mainly due to its complex and polyploid genome. Polyploidy, in fact, on the one hand confers a high degree of plasticity to the organism, on the other hand it makes genetic analysis more difficult for scientists and breeders aimed at dissecting the molecular basis underlying quantitative and qualitative traits (Dubcovsky et al., 2007; Bevan et al., 2017). So far, most efforts to understand the genetic basis of key traits related to yield, grain quality and adaptability have been made through map-based cloning approaches (summarized by Colasuonno et al., 2021; Soriano et al., 2021; Arrigada et al., 2021). For example, genes involved in vernalization and photoperiod response (Yan et al., 2003, 2004; Díaz et al., 2012), grain protein content (Uauy et al., 2006), grain quality (Jin et al., 2018; Goel et al., 2019; Semagn et al., 2021) stem solidness (Nilsen et al., 2020), male sterility (Ni et al., 2017; Xia et al., 2017), and resistance to fungal diseases (Fu et al., 2009) have been identified and cloned. However, this approach is costly and time-consuming, as multiple steps are required, from developing specific mapping populations, through identifying target loci using co-segregating genetic markers, to sequencing relevant loci.

The availability of the reference genome of the allohexaploid landrace Chinese spring (IWGSC et al., 2018; Zhu et al., 2021) has allowed the application and combination of high-throughput sequencing methods to map, identify and clone candidate genes much faster than in the recent past (Henry et al., 2014; Dong et al., 2020; Martinez et al., 2020; Xie et al., 2020). Genome availability has resulted in a remarkable change in bread wheat genetics, which now has a powerful tool for re-sequencing new accessions and for investigating sequence variations across the entire genome. Despite the availability of a gold-standard reference genome, some target genes can only be found in certain cultivars and be absent in the reference accession. For this reason, several strategies have been successfully developed with the aim of reducing genome complexity and sequencing costs and favoring the discovery of a large number of accession-specific variants in wheat (Krasileva et al., 2017; He et al., 2019). For example, genotyping-by-sequencing (GBS) (Elshire et al., 2011; Poland et al., 2012) has been used in wheat to perform genome-wide association studies (GWAS) and quantitative trait loci (QTL) mapping, as well as to disclose patterns of genetic variation (Bernardo et al., 2015; Juliana et al., 2019; Blackburn et al., 2021). Likewise, whole exome sequencing (WES) allows for the identification of nucleotide variability across the exome, i.e., the exon sequences of all protein-coding genes in a genome (Uavy et al., 2017; Mo et al., 2018; He et al., 2019). WES data of nearly 500 accessions from all over the world has been used to reveal the wheat breeding history (Pont et al., 2019). In a separate study, the exome of ∼900 hexaploid and tetraploid wheat accessions has been selectively captured and sequenced to understand how wild-relative introgression enables adaptation in modern bread wheat (He et al., 2019). WES has also been successfully coupled with bulked-segregant analysis (BSA) to identify candidate genes associated with key agronomic traits, as it dramatically reduces genotyping costs by using selective sampling, and the statistical power in QTL-mapping is comparable to that of full-population analysis (Gardiner et al., 2016; Mo et al., 2018; Martinez et al., 2020). For example, Martinez et al. (2020) combined WES with BSA to map ethylmethanesulfonate mutations and identified a novel allele linked to the wheat ERA8 ABA-hypersensitive germination phenotype. Within this motivating context, this study aimed to determine the efficacy of WES for identifying useful alleles within a recombinant inbred line (RIL) population of 46 individuals plus the two parents selected from a previous work in which the entire RIL population was used to identify genomic regions associated with target traits: plant height (PH), juvenile growth habit (GH), heading date (HD), fertile tiller number (FTN) and total tiller number (TTN) (Vitale et al., 2021). We identified many single nucleotide polymorphisms (SNPs) that were classified based on their genomic location and putative biological effect.

To validate the sequencing results, eight SNP-containing coding sequences were used to develop Kompetitive Allele-Specific PCR (KASP) markers to detect and distinguish specific alleles in the entire population. This resource has been primarily established to be used for allele mining (Kumar et al., 2010) and for BSA. The carefully phenotyped genetic materials can be effectively used to identify QTL and trait-associated genes, develop gene markers, and build genomics-assisted prediction models in bread wheat. Indeed, we believe that data FAIRability is an essential prerequisite to ensure the reuse of data and knowledge for downstream investigations, alone or in combination with newly generated data.

## Methods

### Plant material and DNA extraction

Forty-six individuals, derived from the tails of a bi-parental recombinant inbred line population (RIL_F6:7_), previously studied for five high-correlated morpho-physiological traits (i.e., plant height, juvenile growth habit, total tiller number, fertile tiller number and heading date) (Vitale et al., 2021), were analyzed. The population of 176 RILs was developed from a cross between Lankaodali and Rebelde bread wheat cultivars, using the single seed descent method by advancing random F2 plants to the F6:F7 generation (Vitale et al., 2021). The two parental accessions were characterized by contrasting quantitative and qualitative agronomic traits (Botticella et al., 2018; Vitale et al., 2021).

Lankaodali is an early bread wheat cultivar of Chinese origin with very large kernels, low tillering ability, and poor qualitative attributes. Rebelde is an Italian cultivar with late flowering, small kernels, high tillering ability and excellent grain quality traits. During the 2020–2021 growing season, the leaves of 14-day-old seedlings of each of 48 individuals were collected and ground using liquid nitrogen. DNA was extracted using the Quick-DNA Plant/Seed Miniprep Kit (Zymo Research, United States) according to the manufacturer’s instructions. DNA quality and quantity were estimated using the NanoDrop ND-1000 spectrophotometer (Thermo Scientific, Wilmington, DE, United States) and the Qubit fluorometer (Invitrogen, Carlsbad, CA, United States), respectively.

### Exome capture

In-solution-based hybridization was applied to capture the target loci. Baits (304,327) were designed to specifically capture over 250 Mb of coding DNA sequences (CDS) (myBaits®, Arbor Biosciences, Ann Arbour, MI, United States; http://www.arborbiosci.com). Bait design was based on the Chinese spring wheat genome v1.0 (IWGSC et al., 2018) and was carried out following the manufacturer’s protocol version 3.01 (https://arborbiosci.com/wp-content/uploads/2020/01/myBaitsExpert_WheatExome_Product_Sheet.pdf; accessed 14/07/2022).

As shown in Supplementary Table S1, the baits were evenly distributed along the genome, with the lowest number (10,345 baits) on chromosome 4D and the highest (17,587 baits) on chromosome 2B.

### Illumina sequencing and data processing

The extracted DNA (250 ng −1 μg) was subjected to random mechanical shearing to obtain fragments with an average size of 400 bp. The fragments underwent an A-tailing reaction at 3’ of the blunt**-**end, where barcoded adapters were then ligated. Libraries were paired-end sequenced on an Illumina NovaSeq 6,000 platform. After Illumina sequencing, an average of 153 million raw reads *per* individual were obtained, ranging from 96 M (sample 21) to 230 M reads (sample 31) (Supplementary Table S2). Overall sequencing quality was assessed by FastQC (https://www.bioinformatics.babraham.ac.uk/projects/fastqc/) and reads with a base quality score below 20 (Q < 20) were removed using Trimmomatic (Bolger et al., 2014) (SLIDINGWINDOW = 6). High quality reads were then aligned to the Chinese spring wheat genome v1.0 (IWGSC et al., 2018) with BWA-MEM (Li et al., 2009) (MINIMUMSEEDLENGTH = 19; BANDWIDTH = 100). SAMtools (Heng et al., 2009) were used to convert SAM files to BAM; the latter were processed by the *MarkDuplicates* utility of Picard version 1.109, (http://picard.sourceforge.net), to remove duplicate reads. Reads with mapping quality scores below 30 (Q < 30) were also filtered out. QualiMap (Garcia Alcalde et al., 2009) was used to evaluate the effectiveness of the mapping process: the percentage of mapped reads was greater than 99% for each individual with an average error rate of 0.74% (Supplementary Table S2). The reads-to-genome mapping produced over 55% of on-target reads for all individuals except sample 26, which showed the lowest percentage (∼47%) (Supplementary Figure S1). Hybridization capture methods are known to be prone to off-target enrichment and capture (Kaur et al., 2017) and in polyploid species target specificity and efficiency is affected by the presence of homoeologous sequences (King et al., 2015). This reflects the percentage of on-target reads we have achieved which is consistent with what obtained by King et al. (2015).

**FIGURE 1:**
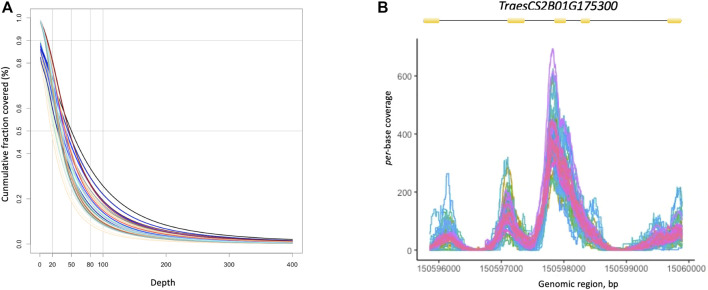
**(A)** Cumulative distribution of coverage depth across target region in 48 wheat individuals. The graph highlights the fraction of bases captured in the target regions covered at a depth ranging between 0x–400x. **(B)** Per-base depth of coverage for the *TraesCS2B01G175300* gene in all individuals. The exon (yellow boxes) -intron (black lines) gene structure is shown above the multi-line graph.

The *CoverageBed* utility of the BEDtools package (http://bedtools.readthedocs.org/) was used to derive the depth of coverage of the target regions for each individual. On average, more than 50% of the bases in the bait regions were covered at a depth greater than 20x (Figure 1A). Coverage *per* individual averaged 13.7x, with most individuals showing a mean coverage between 4x and 35x (Supplementary Table S3). We also estimated the depth of coverage *per*-base at the gene level (Figure 1B). As an example, Figure 1B shows *per*-base depth of coverage for the *TraesCS2B01G175300* gene in all individuals. As expected, the depth of coverage was fairly uniform across exons and very shallow for introns.

### Polymorphism discovery, variant annotation, and biological effect prediction

SNPs were identified using the Genome Analysis Toolkit (GATK) version 4.0 (Van der Auwera et al., 2013), following the best practices recommended by the GATK documentation. The *VariantRecalibrator* utility was used to distinguish true genetic variants from sequencing or data processing artifacts (false positives) and remove the latter from the raw VCF (Variant Call Format) file. A total of 15,046,465 SNPs with a sequencing and alignment quality score ≥30 (Phred-scaled) and a coverage ≥10 were identified. The *VCF-stat* utility in VCFtools (King et al., 2015) was used to retrieve the statistics by sample. The number of variants ranged from 509,707 SNPs (sample 21) to 1,939,789 (sample 37), with a mean of 1,062,065 SNPs. To assess the level of residual heterozygosity for each individual, we calculated the percentage of heterozygosity at each locus by dividing the number of heterozygous genotypes for a given locus by the total number of individuals. This number was averaged across all the chromosomes and returned residual heterozygosity equal to 5.85% (Supplementary Figure S2) **(** Danecek et al., 2011). As expected, transitions (Ts) were the most abundant (∼70%), while transversions (Tv) accounted for ∼30%. Their ratio was 2.11. SnpEff (Cingolani et al., 2012; Velmurugan et al., 2018) was used to classify variants based on genomic location and biological effect (Supplementary Figures S3, S4). As observed by Suren et al. (2016), we found that some of the variants also came from non-target regions, although bait design was exclusively based on annotated exons. Indeed, most SNPs were detected in the intergenic (47%) and upstream gene regions (17%), while approximately 26% fell within exons (Supplementary Figure S3). A stacked bar chart showing SNPs grouped by their impact (low, moderate, and high) is reported in Supplementary Figure S4. SNPs with “moderate” impact on protein functioning were the most abundant (52%). Variants marked as “low” impact accounted, on average, for 46% of all variants (Supplementary Figure S4). Finally, the variants estimated to have a deleterious impact on gene functioning (i.e., “high” impact) accounted for 2% on average. The latter are to be considered the most interesting variants, since they might determine phenotypes of interest.

### Data validation and quality control

**FIGURE 2:**
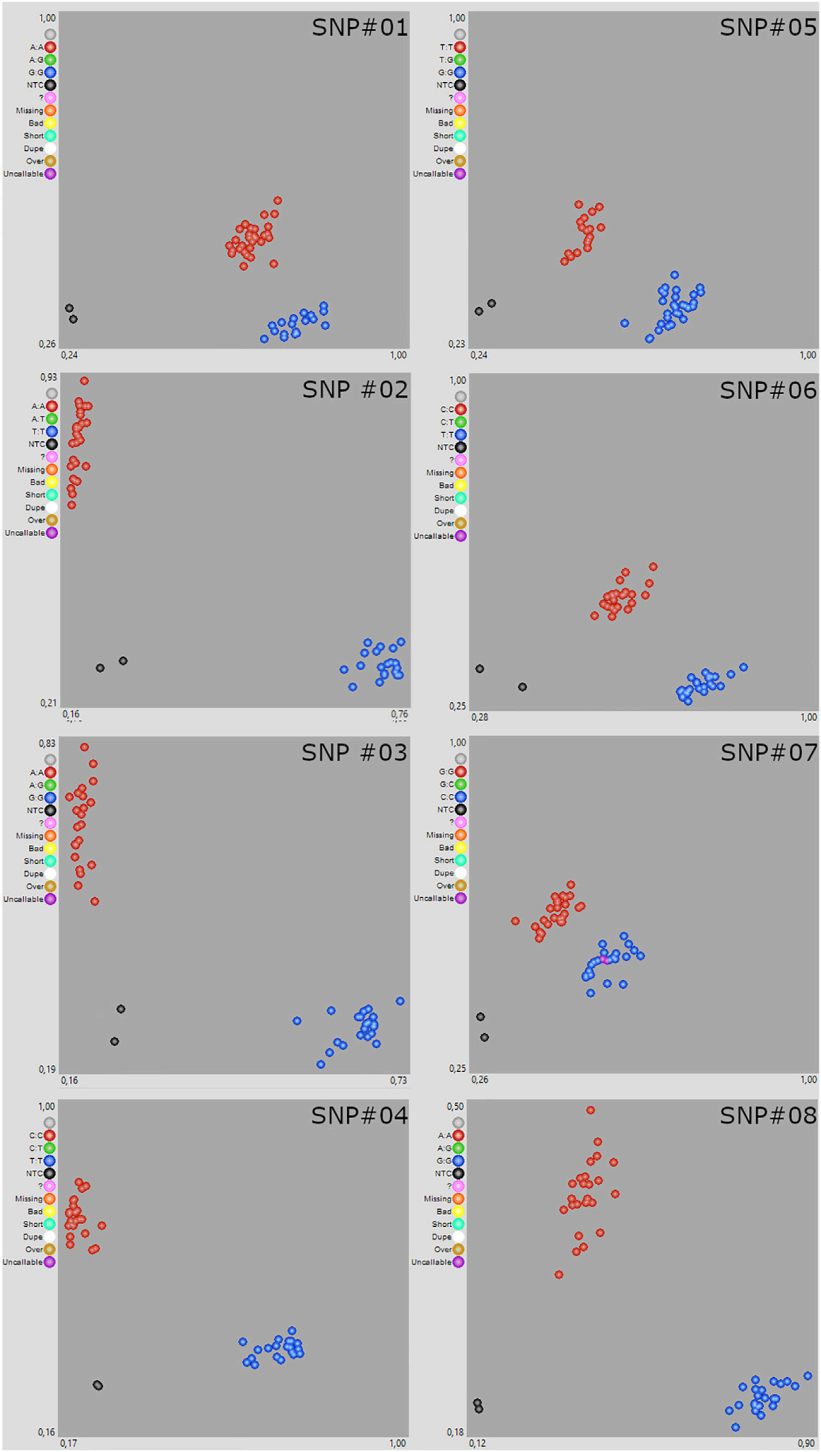
Scatter plot for eight Kompetitive Allele-Specifc PCR (KASP) marker assays in 48 wheat varieties. KASP assays showing clustering of individuals on the X-(FAM) and Y-(HEX) axes. Red individuals have the HEX-type allele; blue individuals have the FAM-type allele. In both cases, individuals are homozygous for the reference or alternate allele. Green individuals are heterozygous for the allele. Black dots represent negative control and pink dots uncallable genotypes.

The conversion of SNP markers into KASP markers is particularly challenging in polyploid crops, due to the presence of homoeologous sequences (Makhoul et al., 2020). To validate the quality of polymorphism discovery and variant annotation generated by exome capture, we designed and tested eight KASP assays that distinguished between alleles for the 48 individuals (Figure 2). SNP markers were selected following different criteria: presence of polymorphism between parental lines, sequencing depth, biological effect, randomness. The KASP primers design was carried out using the commercial KASP assay design service (KASP-by-Design) developed by LGC Biosearch Technologies. For each SNP, 100-bp flanking regions were obtained from the reference genome and converted into KASP to detect the specific parental allele. The LGC Genomics (Hoddeson, United Kingdom) designed two allele-specific forward primers carrying the standard FAM- or VIC-compatible tail (FAM: 5′-GAA​GGT​GAC​CAA​GTT​CAT​GCT-3′; VIC: 5′-GAA​GGT​CGG​AGT​CAA​CGG​ATT-3′) with the targeted SNP at the 3′ end (Supplementary Table S4). Almost all primer pairs had perfect match with the target sequences and mismatches at 3’ tail with the other wheat sub-genomes. DNA samples were arrayed into a 96-well PCR plate, each containing ∼5 µl reaction mix (45 ng of dry DNA, 2.5 µl of 1 × KASP master mixture, and 0.1 µl of primer mix). Primer mix included a final concentration of 30 µM of the common primer and 10 µM of each tail primer. PCR experiments were performed using the ABI ViiA7 instrument (Applied Biosystems, Foster City, CA, United States) as follows: initial denaturation at 94°C for 15 min, followed by ten touchdown cycles (94°C for 20 s; touchdown at an initial temperature of 61°C and decreasing by −0, 6°C *per* cycle for 60 s) reaching a final annealing temperature of 55°C, followed by 26 additional annealing cycles (94°C for 20 s; 55°C for 60 s). Fluorescence readings were performed with a temperature below 37 °C and allelic discrimination plots were drawn using the SNP viewer software (https://www.biosearchtech.com/support/tools/genotyping-software/snpviewer). Individuals with contrasting alleles at each SNP locus (0/0 corresponds to the reference allele, 1/1 to the alternative allele, 0/1 to a heterozygous locus, and “N/A” to a missing data point) were genotyped using the KASP assays (Supplementary Table S5). Results showed that roughly 80% of alleles for all loci were scored identically between WES data and KASP genotyping, including those with low coverage (Supplementary Table S5).

### Potential reuse

In addition to sequence data and sequence variations, we will make available the plant material described in this paper under a standard material transfer agreement This will allow the scientific community to identify marker-trait associations linked to key agronomic traits (including those mentioned above) assessed in other environments (year, site, management), and, thus, detect stable QTL based on genotype-environment (GxE) interactions.

Indeed, SNP discovery supports the use of bulked segregant analysis as a powerful tool to accelerate gene identification and QTL mapping cost-effectively.

In addition, the data described here could be used to dissect naturally occurring allelic variation at candidate genes controlling key agronomic traits, identify useful alleles (i.e., loci affecting the traits of interest) and understanding their function.

## Data Availability

The datasets presented in this study can be found in online repositories. The names of the repository/repositories and accession number(s) can be found below: https://www.ncbi.nlm.nih.gov/, Accession Number PRJNA821683; https://figshare.com/, DOI: https://doi.org/10.6084/m9.figshare.19485569.v1.
