# Supplementary material for: Whole-exome sequencing of selected bread wheat recombinant inbred lines as a useful resource for allele mining and bulked segregant analysis
Source: Front Genet. 2022 Nov 22;13:1058471. doi: 10.3389/fgene.2022.1058471 (PMC9723387; doi:10.3389/fgene.2022.1058471)
Supplement: Supplementary file 3 [file Table1.DOCX]

| **Chromosome** | **# baits** | **Average bait length (bp)** |
| --- | --- | --- |
| 1A | 12,426 | 749.51 |
| 2A | 16,870 | 726.78 |
| 3A | 14,626 | 775.07 |
| 4A | 14,173 | 724.99 |
| 5A | 15,082 | 784.44 |
| 6A | 11,872 | 735.11 |
| 7A | 15,207 | 737.71 |
| Genome A | 100,256 | 747.71 |
| 1B | 13,682 | 719.34 |
| 2B | 17,587 | 726.28 |
| 3B | 16,498 | 748.22 |
| 4B | 11,297 | 785.85 |
| 5B | 16,226 | 748.97 |
| 6B | 13,415 | 713.04 |
| 7B | 13,715 | 730.06 |
| Genome B | 102,420 | 737.82 |
| 1D | 12,806 | 767.13 |
| 2D | 17,022 | 732.08 |
| 3D | 14,748 | 801.93 |
| 4D | 10,345 | 817.46 |
| 5D | 15,178 | 825.84 |
| 6D | 11,199 | 814.66 |
| 7D | 14,407 | 826.58 |
| Genome D | 95,705 | 795.52 |

**Supplementary Table S1.** Chromosomal distribution of the baits. The number of baits per chromosome and their mean length (bp) are listed.
