# Supplementary material for: Whole-exome sequencing of selected bread wheat recombinant inbred lines as a useful resource for allele mining and bulked segregant analysis
Source: Front Genet. 2022 Nov 22;13:1058471. doi: 10.3389/fgene.2022.1058471 (PMC9723387; doi:10.3389/fgene.2022.1058471)
Supplement: Supplementary file 4 [file Presentation1.PPTX]

## Slide 1
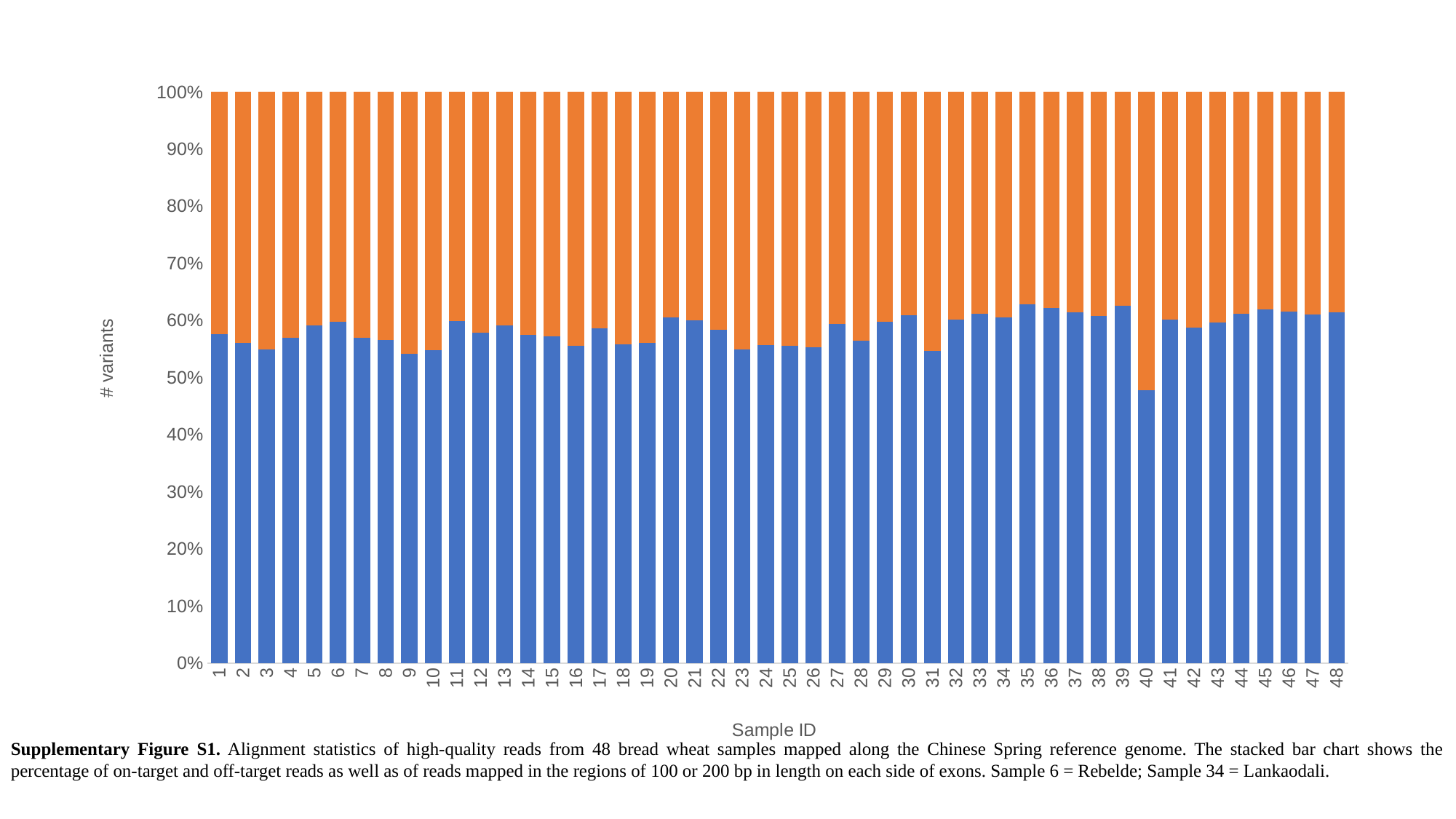

### Chart
| Category | HOM | HET |
|---|---|---|Supplementary Figure S1. Alignment statistics of high-quality reads from 48 bread wheat samples mapped along the Chinese Spring reference genome. The stacked bar chart shows the percentage of on-target and off-target reads as well as of reads mapped in the regions of 100 or 200 bp in length on each side of exons. Sample 6 = Rebelde; Sample 34 = Lankaodali.
