# Supplementary material for: Whole-exome sequencing of selected bread wheat recombinant inbred lines as a useful resource for allele mining and bulked segregant analysis
Source: Front Genet. 2022 Nov 22;13:1058471. doi: 10.3389/fgene.2022.1058471 (PMC9723387; doi:10.3389/fgene.2022.1058471)
Supplement: Supplementary file 5 [file Presentation4.PPTX]

## Slide 1
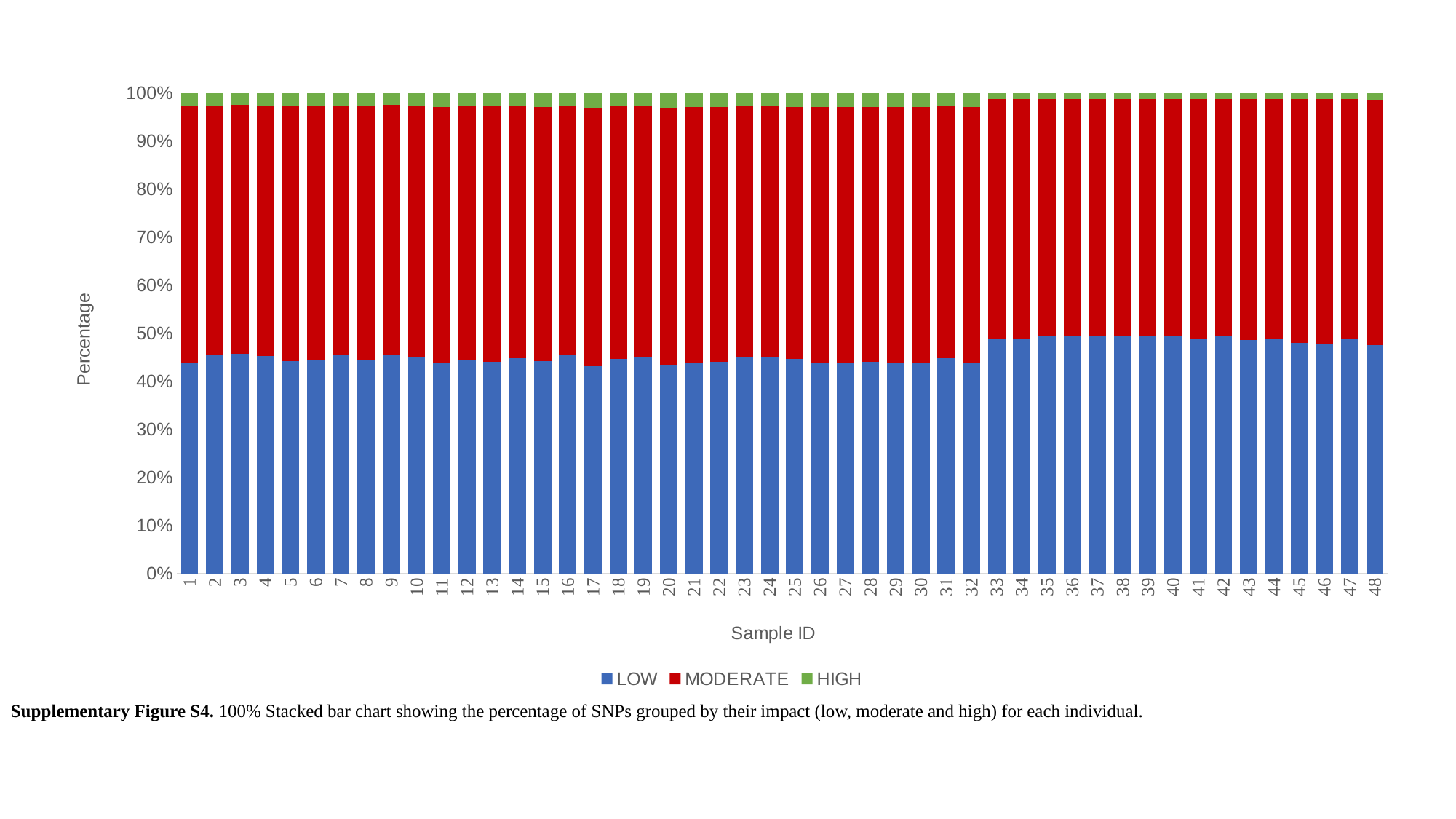

### Chart
| Category | LOW | MODERATE | HIGH |
|---|---|---|---|Supplementary Figure S4. 100% Stacked bar chart showing the percentage of SNPs grouped by their impact (low, moderate and high) for each individual.
