# Supplementary material for: Whole-exome sequencing of selected bread wheat recombinant inbred lines as a useful resource for allele mining and bulked segregant analysis
Source: Front Genet. 2022 Nov 22;13:1058471. doi: 10.3389/fgene.2022.1058471 (PMC9723387; doi:10.3389/fgene.2022.1058471)
Supplement: Supplementary file 7 [file Presentation3.PPTX]

## Slide 1
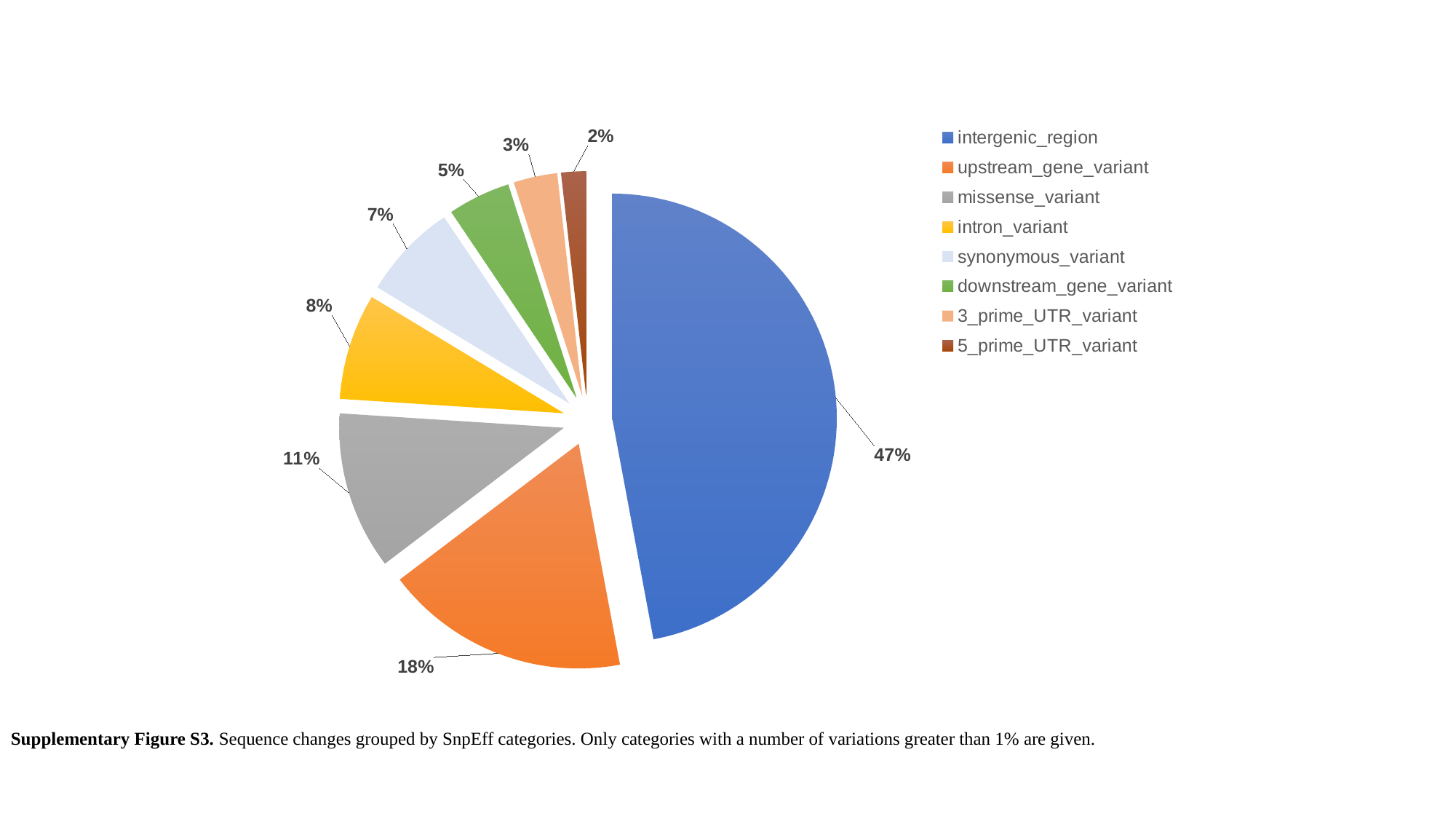

### Chart
| Category | | |
|---|---|---|
| intergenic_region | 6777476.0 | 45.04364014779284 |
| upstream_gene_variant | 2536218.0 | 16.855904901523054 |
| missense_variant | 1635941.0 | 10.872592939764061 |
| intron_variant | 1098958.0 | 7.303761561020375 |
| synonymous_variant | 994634.0 | 6.610416027258494 |
| downstream_gene_variant | 649120.0 | 4.314102726846291 |
| 3_prime_UTR_variant | 452500.0 | 3.0073506961701173 |
| 5_prime_UTR_variant | 258244.0 | 1.7163099959817807 |Supplementary Figure S3. Sequence changes grouped by SnpEff categories. Only categories with a number of variations greater than 1% are given.
