# Supplementary material for: Whole-exome sequencing of selected bread wheat recombinant inbred lines as a useful resource for allele mining and bulked segregant analysis
Source: Front Genet. 2022 Nov 22;13:1058471. doi: 10.3389/fgene.2022.1058471 (PMC9723387; doi:10.3389/fgene.2022.1058471)
Supplement: Supplementary file 8 [file Presentation2.PPTX]

## Slide 1
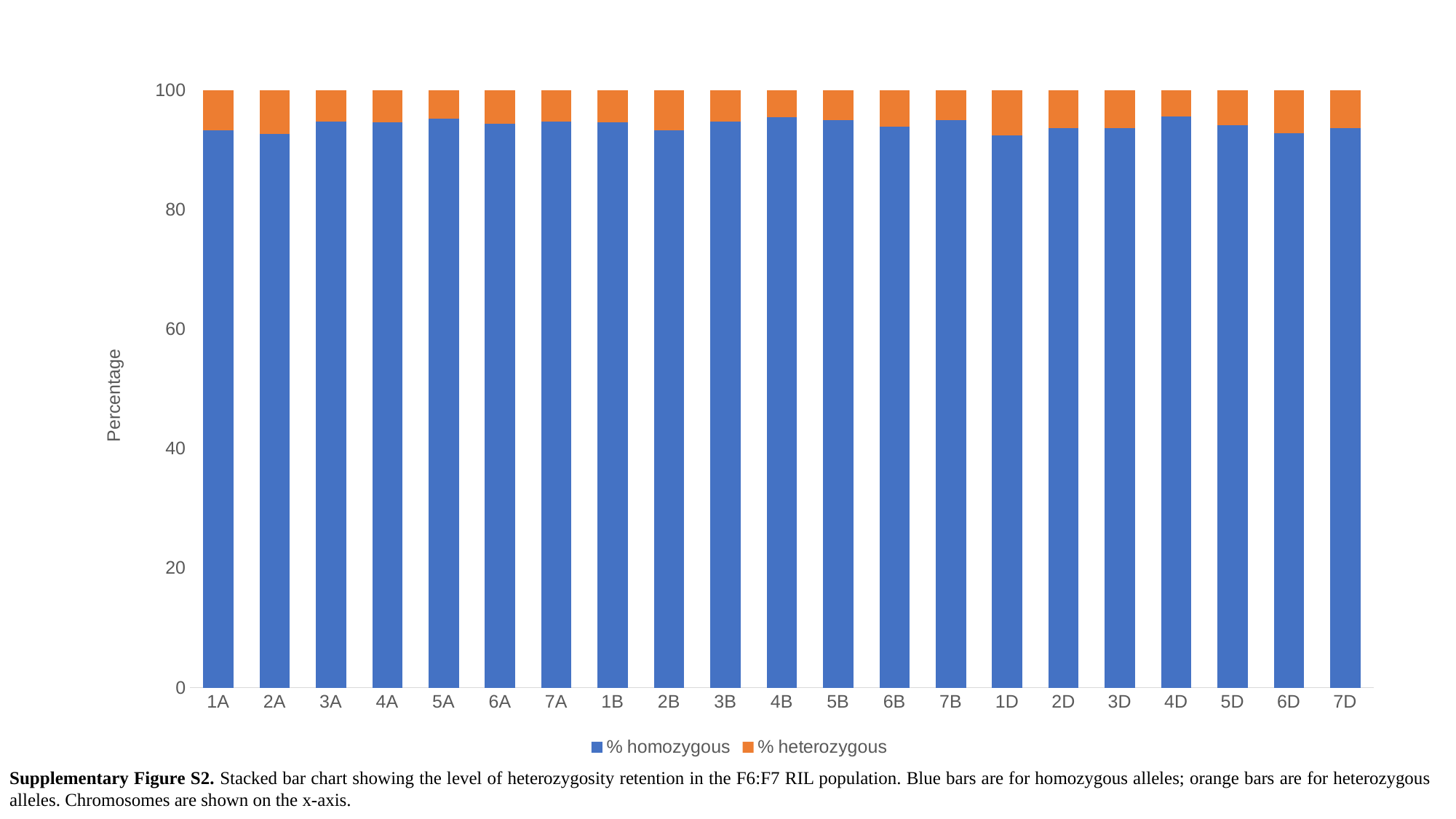

### Chart
| Category | % homozygous | % heterozygous |
|---|---|---|
| 1A | 93.23405 | 6.76595 |
| 2A | 92.69574 | 7.30426 |
| 3A | 94.67487 | 5.32513 |
| 4A | 94.60021 | 5.39979 |
| 5A | 95.1702 | 4.8298 |
| 6A | 94.34164 | 5.65836 |
| 7A | 94.66764 | 5.3323599999999995 |
| 1B | 94.55786 | 5.44214 |
| 2B | 93.29955 | 6.700449999999999 |
| 3B | 94.75953 | 5.24047 |
| 4B | 95.41549 | 4.58451 |
| 5B | 94.99983 | 5.000170000000001 |
| 6B | 93.85283 | 6.14717 |
| 7B | 95.02336 | 4.976640000000001 |
| 1D | 92.42515 | 7.57485 |
| 2D | 93.66427 | 6.335730000000001 |
| 3D | 93.60564 | 6.394360000000001 |
| 4D | 95.62845 | 4.37155 |
| 5D | 94.10553 | 5.89447 |
| 6D | 92.76173 | 7.238269999999999 |
| 7D | 93.67032 | 6.32968 |Supplementary Figure S2. Stacked bar chart showing the level of heterozygosity retention in the F6:F7 RIL population. Blue bars are for homozygous alleles; orange bars are for heterozygous alleles. Chromosomes are shown on the x-axis.
